# Pennsylvania State Dental Society

**Published:** 1886-11

**Authors:** Wm. H. Trueman


### PENNSYLVANIA STATE DENTAL SOCIETY.

EIGHTEENTH ANNUAL MEETING, HELD AT CRESSON, JULY 27, 28 AND 29, 1886.

REPORTED FOR THE INDEPENDENT PRACTITIONER, BY WM. H. TRUEMAN, D. D. S.

Concluded from page 567.

### WEDNESDAY AFTERNOON SESSION.

A portion of the time was devoted to clinics and to the examination of new instruments and appliances.

Dr. Hamlin Barnes demonstrated the use of his prepared gold for lining vulcanite plates. He urged, where the lining is used, the importance of having, to vulcanize upon, a cast with a surface smooth and free from air bubbles. To obtain this he preferred to prepare the impression by giving it several coats of thin sandarach varnish. If the impression is made in modelling compound, the varnishing must be done quickly or the alcohol may soften the surface. He prepares the plaster for casting the impression by first placing sufficient water in a bowl; into this the plaster is sifted, a little at a time, from a scoop, the bottom of which is made of fine wire gauze, allowing it to settle after each addition. Sufficient plaster having been added, it is allowed to settle, the surplus water poured off and the thin batter poured into the impression, until the face of it is covered; this is jarred to cause it to flow into the depressions, and after it has slightly hardened a batter of greater consistency is added to make the cast of sufficient thickness. He used a slow setting plaster, necessarily, as by this method considerable time is required. The surface of the cast made at the clinic was remarkably smooth and free from air bubbles, but when examined the next day, was too soft for practical use in the laboratory; evidently too much time had been consumed in mixing the plaster, or the batter was too thin to produce a hard, serviceable cast. This might be due, however, to interruptions incident to a clinical demonstration.

The case is flasked and packed in the usual way; to prevent the rubber adhering to the cast, it is covered with an alloy of lead and cadmium rolled to about No. 40, and to facilitate opening the flask, during the process of packing, a piece of thin muslin is placed between the rubber and the cast; this is removed before the flask is finally closed. After the flask is closed, and when in the usual method of procedure it would be ready to be placed in the vulcanizer, the muslin having been removed but the lead foil still remaining in position, it is allowed to cool, preferably in the air, but if time is an object, by immersing in cold water. When quite cold the flask is opened, the lead foil stripped off, any surplus rubber cut off with a pair of scissors, and the prepared gold applied, either to the surface of the rubber or to the cast. In either case it is cut into pieces of suitable size and shape to fit neatly, allowing the edges barely to lap; if the pieces lap too much the edges are liable to rise after the case has been worn, and thus form a rough line; the gold is "patted" into close contact with the rubber, and after the surface is entirely covered the flask is closed and the case vulcanized. As the gold is a trifle thinner than the lead foil, it is better not to quite close the flask until the gold is placed in position. Particular stress was laid upon allowing the flask and its contents to cool before opening to insert the gold. If the flask is opened immediately, there is a tendency in the rubber to draw away from the mould. From this cause, when the flask is again closed, the position of the gold is likely to be changed, the joints between the pieces may be opened, it may be thrown into folds, or even torn over depressions, as was shown by illustrations. The speaker suggested that it would be better, in cases in which tin foil was used simply to give a clean smooth surface to the palatal portion of the plate, to adopt the same plan and defer placing the tin foil in place until the flask had cooled. Care is, of course, needed, to avoid injury to the cast if any portions are weak.

The Partz Electric Battery Company, of Philadelphia, exhibited a new form of battery known as the "Acid Gravity Battery," specially designed for office use. In construction it closely resembles the well known "Gravity Battery." Each cell is composed of an oblong glass vessel seven and one-half inches long; five and one-half inches wide, and seven and one-half inches high. A carbon plate, the upper surface of which is covered with cone-shaped projections so as to present a more extended surface, fits the bottom of the cell, and into the middle of one end of this is fitted, by grinding, a carbon rod, extending above the top of the cell and forming one pole of the battery. About two inches above the carbon plate is suspended, horizontally, a cast zinc plate; a glass tube, funnel-shaped, extends above the top of the cell, the narrow end, through which there is a small hole, resting upon the carbon plate at the end opposite to the carbon rod. The cell is closed by a wooden cover, the carbon rod, an extension of the zinc plate, and the glass tube passing through suitable openings and projecting above it. The battery is charged with a solution of either an alkaline sulphate, preferably that of magnesia (one pound to two quarts of water), or an alkaline chloride, preferably that of ammonium (one pound to five pints of water), each cell requiring about forty-five fluid ounces. The solution of sulphate of magnesia, while not giving as strong a current as the other salts, is preferred on account of its cheapness and cleanliness. After the cell is charged, a small quantity of salt produced by causing sulphuric and chromic acid to combine, forming an amorpho-crystalline mass, known as "sulpho-chromic salt" (made and furnished by the company), is dropped into the glass tube. In a short time this is dissolved, and the resulting solution having a much greater specific gravity than the solution filling the cell, spreads over and covers the carbon plate. The battery then becomes active and continues so as long as any of this remains. When the battery becomes weak, it is only necessary to add a fresh portion of the salt, dropping it into the glass tube. Four cells operate the electric mallet effectively. The advantages claimed for the battery are its cleanliness, entire absence of any fumes, the non-corrosive character of the solution used, and the ease with which it is kept in working order. The cells do not require cleaning oftener than once in two or three months, the addition of a small portion of the sulpho-chromic salt once a week being sufficient to keep it in order. There is an entire absence of the "chrome alum" deposit, so annoying with the ordinary carbon or Bunsen Battery. Dr. Guilford reported that he had been using four cells to operate an electric mallet, his door bell, and an electric gas-lighting apparatus, for about nine months, during which time the solutions had been changed but once, the only attention it had received being the addition of the sulpho-salt from time to time.

EVENING SESSION.

Dr. C. S. Beck, in relating various incidents of practice, expressed the opinion that a large portion of capped pulps, where there had been actual exposure, especially if that exposure was caused directly by decay, would eventually die. Whenever possible he preferred not to expose a pulp; the softened dentine makes by far the best cap. He had been very much interested and impressed by the recent investigations of Drs. Miller, Black, and others, upon the germ theory and its bearing upon dental caries, and also the part assigned to micro-organisms in causing and maintaining pathological conditions. This had led him to abandon all escharotics in these cases, and to use bichloride of mercury in solutions of various strength, from one that is almost saturate to that of about one grain to the ounce. He used this in various suppurative conditions, and applied it freely to the cavities where, to avoid pulp exposure, he allowed more or less softened dentine to remain. He used a rather strong solution, as he desired to destroy, not only any germs that may be present, but to thoroughly sterilize the parts so that they could not in the future become the home of these destructive organisms.

Dr. Guilford called attention to the strength of the solution of bichloride of mercury recommended; he thought it entirely too strong to be safely used. One part in ten thousand of water was an effective germicide; then why use it so strong as one part in five hundred ? It is a most violent poison, and should be used with the greatest care.

Dr. Jack was more hopeful of the result of pulp capping. He had found deposits of secondary dentine in a notable number of cases, and under a variety of methods of treatment. Accidental exposures were far more amenable to treatment. He had treated some cases of quite extensive exposure that had given no trouble. In considering this question we should distinguish cases of mere exposure from cases of diseased pulps, as in the latter, failure was far more frequent. We there labor under a serious difficulty in not knowing the exact condition, or how far the pathological changes may have advanced.

Dr. Klump, in all cases where the exposure was the direct result of caries, preferred thoroughly to expose the pulp. He thought it advantageous to do so for the reason that, in some cases where he had least expected it, after fully exposing he had found the pulp almost putrescent. Where vitality still remains, if the softened dentine is entirely removed and the pulp fully exposed and carbolized, it will prove successful in nine cases out of ten.

Dr. Guilford said a pulp will not live after any great mechanical or medicinal injury. We should be careful not to apply too strong a remedy. The pulp will not tolerate any space between itself and the capping. Should any exist, it will spread out and strangulate the capillaries upon the rough edges. A cap should be close fitting, non-irritating, and thoroughly protective. He thought Dr. Klump's idea a great mistake; nature's cap is by far the best, and softened dentine, sterilized, forms the best protection.

### CAVITY LINING.

Dr. S. B. Luckie, of Chester, read a paper having this title. He said that the operation of lining a cavity consists in covering the walls with a layer of some material that will subserve a purpose not expected of the filling. Fillings of gold and of amalgam are more durable than those made of the cements, yet the latter will often preserve a tooth, even after a large portion of the filling has been dissolved or worn away by mechanical forces. The deduction then is that the cements, although more easily destroyed by the destructive agents that come in contact with them, are better tooth conservators than the metals, and that more promising results might be obtained by the conjoined use of both in the same cavity.

Large and deep cavities may be partially filled with one of the zinc cements, a metallic filling being placed over and well anchored into it. In this way the effects of thermal shock may be modified, frail walls supported, discoloring of tooth substance prevented, and the objectionable showing of fillings through transparent walls avoided. In selecting materials for cavity lining, their respective properties should be considered, otherwise the object desired may be defeated. Oxy-chloride of zinc, from its stimulating properties, is desirable for lining teeth of poor structure which contain vital pulps. Its power to stimulate activity in the nutritive currents will produce an excess of the pabulum which nourishes and organizes dentine. On the other hand, if used in excess, or without a protection to guard its initiatory effects upon the pulp, the result will be congestion and death of that organ. The class of teeth in which the danger from chloride of zinc is greatest are those denominated soft, low grade, poor structure, etc. It is in these, also, that its exciting qualities should be most effective for good.

That the stimulating effect may be held in check, exposed or nearly exposed pulps should be protected by some form of cap. A concave disk of tin, readily made at the time it is to be used, answers very well for this purpose if the cavity be filled with a paste of oxide of zinc and oil of cloves or creosote. If it is carefully placed in position, it not only protects the pulp from pressure, but modifies to a great extent the physiological action of the cement. Where the cavity does not approach the pulp the disk may be omitted, the paste of zinc oxide and creosote being a sufficient protection.

When the object is simply to prevent a filling showing through thin walls, and the tooth is of a markedly positive color, the cement may be shaded with a suitable pigment so as to more nearly harmonize with it. Phosphate of zinc possesses attributes that make it a better lining, from a mechanical standpoint, than the oxychloride; its ductility allows it to be placed against frail walls with a burnisher instead of cotton pellets, leaving a smooth surface; it is non-shrinking, hardens quickly, and has greater edge strength. It can also be used with safety in teeth with vital pulps; he deemed it important, however, that a protecting layer of some material be placed between it and the pulp in all deep cavities, for although the phosphoric acid may not interfere with the nutrition of the pulp, the pressure necessary for its adaptation might be sufficient to cause congestion.

The advantages of lining cavities with the cements named are, briefly stated, less liability of recurrence of decay, color of tooth sustained when amalgam is used, discolored teeth improved in color, thermal shock modified, and liability of fracture reduced. In cases where the cements are undesirable, thick sandarach varnish, or gutta-percha dissolved in chloroform, has been recommended, and will often be of excellent service.

Dr. Magill doubted whether chloride of zinc, when combined with the oxide as it is in the oxy-chloride of zinc cement, possesses the therapeutic value suggested by the essayist. The mechanical protection it affords can be secured equally well with a far less irritating cement; this being the case, what is gained if all the decay is removed from the cavity, by using chloride of zinc to counterbalance the risk of its causing serious irritation? If we could accurately diagnose the condition of the pulp, we might in some cases derive benefit from its use, but this we cannot well do. The great advantage of cavity lining, to his mind, is the economy of more expensive material, the saving of time and effort to the operator, and of time, money, and pain to the patient.

Dr. R. B. Cummins, of Blairsville, read a paper entitled

A STUDY IN DENTISTRY.

He considered the subject from the standpoint of a country dentist, and regretted that his isolated position deprived him of that spur or incentive to put forth his best efforts which the competition and association of a large community develops in his city brother. This frequently causes the rural practitioner to fall into ruts, and to conduct his business by routine methods. He referred to the importance of keeping the instrument case and the instruments clean and in good order, the importance of instructing patients in the proper use of tooth-powder and the tooth-brush, etc.

Dr. Magill, referring to the remark in the paper about country dentists falling into ruts, said the question with him was whether we do not all of us fall into ruts. The tendency seems to be to get down to the level of our surroundings. He knew many dentists living in little country towns who had kept well up with the times; they have resisted the temptation to step down to the level of those around them, their ambition having led them to strive to work up. They make excellent fillings, pride themselves upon contour work, use the electric mallet, skillfully insert crowns, and by close attention to business and a thorough devotion to their profession, in many cases build up an excellent and appreciative practice. They gather around them patients who appreciate and are willing to pay as good prices for good work as the city patient.

THURSDAY MORNING.

Dr. W. E. Van Arsdel, of Sharon, read a paper upon "All Sorts of Questions."

He said his field for practice is in a district full of malaria, where quinine is an article of regular diet. He had seen numerous instances where, after the insertion of small fillings in teeth in which no after-trouble should be anticipated, an attack of malaria seemed to be the inciting cause of very decided trouble; in some cases periostitis, in others death of the pulp and alveolar abscess. Can anything be done to avoid such results? His experience in capping pulps has, for the same reason, been decidedly unsatisfactory, and seems to be but the sure precursor of death. He related the case of a patient whose teeth, originally strong, were being rapidly destroyed, owing, he thought, to the free and constant use of candy. He was a manufacturer of candy, and ate probably a pound a day. He asked what could be done to stop their rapid decay. He had advised the local application of prepared chalk. Is there anything better than that?

Dr. Beck stated that, in cases where from long continued use the system had become so saturated with quinine that it seemed to have lost its effect, he had excellent results from the cautious use of arsenic, in the form of Fowler's solution.

After the transaction of the routine business, the newly elected President, Dr. E. P. Kremer, was conducted to the chair. He appointed the following committees:

*Executive Committee*—C. S. Beck, Chairman, Wilkesbarre; J. C. M. Hamilton, Secretary, Tyrone; G. W. Klump, W. H. Trueman, S. H. Guilford.

*Publication Committee*—W. H. Trueman, Chairman, Philadelphia; S. H. Guilford, Secretary, Philadelphia; E. T. Darby, Alonzo Boice, J. R. C. Ward, W. B. Miller, J. C. M. Hamilton.

*Committee on Enforcement of Dental Law*—W. E. Magill, J. W. Rhone, J. C. Green.

*Committee on Legislative Action*—C. N. Pierce, G. W. Klump, W. E. Magill, H. Gerhart, J. P. Thompson.

Adjourned to meet at Glen Summit, on the last Tuesday in July, 1887.

